# Unvalidated Cerebrospinal Fluid Pressure Equations Should not Be Used for Research

**DOI:** 10.18502/jovr.v17i4.12343

**Published:** 2022-11-29

**Authors:** David Fleischman, Hanspeter E. Killer

**Affiliations:** ^1^Department of Ophthalmology, University of North Carolina at Chapel Hill, Chapel Hill, North Carolina, USA; ^2^Kantonsspital Aarau, Department of Ophthalmology, Aarau, Switzerland; ^4^David Fleischman: https://orcid.org/0000-0002-1043-2021

## Dear Editor,

We have found an unsettling trend of articles being published with the use of an unvalidated formula for estimating cerebrospinal fluid pressure. The study, “Translaminar Pressure Difference and Ocular Perfusion Pressure in Glaucomatous Eyes with Different Optic Disc Sizes'^[1]^ by Cruz and colleagues has a very intriguing hypothesis. However, the authors did not do their due diligence in confirming the origin of the estimation equation central to their investigation. They erroneously attributed the formula to Xie et al's study published in *Critical Care*, 2013.^[2]^ Biophysical parameters including, importantly, the anatomic marker of width of the orbital cerebrospinal fluid space, were used to devise their estimation equation. The equation utilized in Cruz's study, although found in other similarly flawed investigations, has not been validated.

The use of estimation equations in general have been found to poorly represent CSF pressure.^[3]^ Therefore, a parameter that is already exquisitely difficult to measure, perioptic subarachnoid space cerebrospinal fluid pressure, will be further confounded by using these faulty data. To substantiate the concept of the translaminar pressure gradient as a mechanism involved in the pathophysiology of glaucoma, we need robust data. Flawed approximation of CSF pressure is in no way helpful in advancing the science in glaucoma research.

We obviously encourage further study into the relationship between cerebrospinal fluid and ophthalmic disease, but we must all be diligent to prevent further use of unvalidated methods infiltrating this field.

##  Financial Support and Sponsorship

None.

##  Conflicts of Interest

There are no conflicts of interest.

## References

[B1] Cruz NF, Santos KS, Matuoka ML, Kasahara N (2021). Translaminar pressure difference and ocular perfusion pressure in glaucomatous eyes with different optic disc sizes. J Ophthalmic Vis Res.

[B2] Xie X, Zhang X, Fu J, Wang H, Jonas JB, Peng X, et al (2013). Noninvasive intracranial pressure estimation by orbital subarachnoid space measurement: The Beijing Intracranial and Intraocular Pressure (iCOP) study. Crit Care.

[B3] Fleischman D, Bicket AK, Stinnett SS, Berdahl JP, Jonas JB, Wang NL, et al (2016). Analysis of cerebrospinal fluid pressure estimation using formulae derived from clinical data. Invest Ophthalmol Vis Sci.

